# Microparticles: A New Perspective in Central Nervous System Disorders

**DOI:** 10.1155/2014/756327

**Published:** 2014-04-09

**Authors:** Stephanie M. Schindler, Jonathan P. Little, Andis Klegeris

**Affiliations:** ^1^Department of Biology, University of British Columbia Okanagan Campus, 3333 University Way, Kelowna, BC, Canada V1V 1V7; ^2^Health and Exercise Sciences, University of British Columbia Okanagan Campus, Kelowna, BC, Canada V1V 1V7

## Abstract

Microparticles (MPs) are a heterogeneous population of small cell-derived vesicles, ranging in size from 0.1 to 1 **μ**m. They contain a variety of bioactive molecules, including proteins, biolipids, and nucleic acids, which can be transferred between cells without direct cell-to-cell contact. Consequently, MPs represent a novel form of intercellular communication, which could play a role in both physiological and pathological processes. Growing evidence indicates that circulating MPs contribute to the development of cancer, inflammation, and autoimmune and cardiovascular diseases. Most cell types of the central nervous system (CNS) have also been shown to release MPs, which could be important for neurodevelopment, CNS maintenance, and pathologies. In disease, levels of certain MPs appear elevated; therefore, they may serve as biomarkers allowing for the development of new diagnostic tools for detecting the early stages of CNS pathologies. Quantification and characterization of MPs could also provide useful information for making decisions on treatment options and for monitoring success of therapies, particularly for such difficult-to-treat diseases as cerebral malaria, multiple sclerosis, and Alzheimer's disease. Overall, studies on MPs in the CNS represent a novel area of research, which promises to expand the knowledge on the mechanisms governing some of the physiological and pathophysiological processes of the CNS.

## 1. Introduction 

The central nervous system (CNS) is composed of complex cellular networks made up predominantly by neurons and glia (astrocytes, oligodendrocytes, and microglia), which are cells that provide support and protection for neurons [1]. The CNS cells are in close contact with endothelial cells that control blood flow and form the blood-brain barrier (BBB), which in turn is important for controlling the transport of nutrients and macromolecules into and out of the brain [2]. Due to the BBB's role as a physical barrier, the CNS was long considered to be an “immune privileged” site, devoid of any immune cells, and essentially “invisible” to immune system.

Over the years accumulating evidence, however, has shown that the CNS can be invaded by immune cells, which then mount an immune response. Therefore, the term “immune privileged” has now been replaced with “immune specialized”. The brain and the blood stream are in a constant state of bidirectional exchange of cells and macromolecules in order to maintain brain integrity and homeostasis and to allow for repair by immune cells upon injury [1]. This neuroimmune exchange occurs mainly at the level of the neurovascular unit, which is composed of endothelial cells, pericytes, neurons, and glia.

CNS cells can be subjected to a variety of stressors (e.g., toxins, oxygen radicals, and inflammatory mediators), which can change the immune status of the CNS [3]. Consequently, under certain pathological conditions in which the brain microenvironment is altered due to disease-induced stress, infections, or trauma, the injured CNS becomes immune competent and immune reactive [1]. Immune responses in the CNS may be directed against a self or non-self antigen and can involve cellular and molecular pathways that rely on cell-cell communication. During the last few years, studies have revealed extracellular membrane vesicles as new specialized structures for intercellular communication [4–6]. Moreover, it is becoming increasingly evident that these vesicles may be linked to the onset and progression of a variety of diseases including cancer, inflammatory, autoimmune, and cardiovascular conditions, as well as CNS pathologies, which will be discussed in detail in this review.

First, it is necessary to define these particular membrane vesicles, as there has been some debate on the terms used to describe them [7]. Some studies refer to them as microparticles (MPs) [8], microvesicles [9], or ectosomes [10]. Moreover, some researchers use the term microvesicles to describe both MPs and exosomes [11], which is another type of secreted vesicle. In addition, in the biomedical literature, the term “microparticle” is sometimes used to describe the biopolymer particles used as drug delivery systems. In order to avoid ambiguity, in this review, the membrane vesicles of interest will be referred to as microparticles or MPs for short.

Wolf first described MPs, in 1967, in association with platelets in human plasma. As a result, they were termed “platelet dust” and were thought to be inert by-products of platelet activation [12]. Recent research, however, has discovered that MPs are, in fact, a heterogeneous population of membrane-derived vesicles that play a role in regulating various biological and physiological processes, including cell-cell communication, cell proliferation, coagulation, and inflammation. MPs can be released by a diverse population of eukaryotic and prokaryotic cells and multicellular organisms upon activation or apoptosis, particularly under conditions of stress or injury. This causes an increase in the intracellular calcium concentration leading to rearrangement of the cytoskeleton, allowing for the budding of MPs directly from the plasma membrane [13, 14]. In addition, MPs have been implicated to have pathological roles in many diseases such as rheumatoid arthritis, vascular diseases, cancer, diabetes, and Alzheimer's disease (AD) [15–18]. In the CNS, MPs have been detected in the cerebrospinal fluid (CSF), where they are released by nearly all cell types [19, 20]. MPs may play both physiological and pathophysiological roles; they have been implicated in neuronal development, synaptic activity, nerve regeneration, and protective mechanisms [21]. MPs are also capable of transferring toxic proteins between cells, which have implications for neurodegenerative disorders such as AD [22]. Elevated levels of MPs have been detected in the CSF and plasma of individuals suffering from multiple sclerosis (MS) [20, 23, 24] and cerebral malaria [25], as well as a variety of other CNS pathologies. The consensus among recent studies is that increased levels of specific types of MPs in plasma and CSF may represent reliable biological markers for the onset and progression of CNS diseases [20, 26, 27].

This review will summarize the current information on MPs, including their cellular shedding mechanisms and their composition, as well as the analytical methods used to isolate them. A particular focus will be on the emerging roles of MPs in the CNS physiology and their contributions to select neurodegenerative and neuroinflammatory disorders.

## 2. Definition and Classification of Membrane Vesicles

Several attempts using different approaches have been made to define the key characteristics of each type of secreted membrane vesicle. The most studied types of membrane vesicles include exosomes, apoptotic bodies, and MPs. Depending on their cellular site of origin, these vesicles have distinct structural and biochemical properties [28], which also affect their function and the roles that they play in biological systems (see Table 1). This review will focus on MPs, which are a heterogeneous population (0.1–1 *μ*m) of membrane vesicles (comparable in size to bacteria and insoluble immune complexes [29–31]). The biogenesis of MPs is one of the main factors that distinguishes them from other membrane vesicles.

Although MPs can be released during apoptosis (programmed cell death), they differ from apoptotic bodies. After the initiation of apoptosis, the cell shrinks and undergoes chromatin condensation followed by cellular rearrangement. Eventually, the apoptotic cell collapses and fragments, releasing membrane-coated vesicles known as the apoptotic bodies [31]. The apoptotic bodies are released at the end of the apoptosis, while MPs are released during the early stages of apoptosis [13]. In addition, apoptotic bodies differ from MPs in size and in composition. Apoptotic bodies on average have a larger diameter [28] than MPs and they contain nuclear material, cellular organelles, and membrane/cytosolic fragments [32, 33]. Similar to MPs, apoptotic bodies externalize phosphatidylserine (PS); therefore, other factors need to be considered when differentiating between these two types of vesicles. In addition to size and composition, protein markers can be used. The main protein markers of MPs are integrins, selectins, and the CD40 ligand [28]. Even though several studies have suggested that histones are reliable protein markers of apoptotic bodies [28, 34, 35], they could also be present, along with DNA, in MPs [14, 36]. Therefore, when differentiating between apoptotic bodies and MPs, it is essential to consider several characteristics of membrane-derived vehicles, such as those outlined in Table 1.

MPs are also biochemically and morphologically distinct from exosomes, another type of membrane vesicle. The latter are on average smaller (30–100 nm) than MPs [37, 38] and overall more homogeneous in their size and composition compared to MPs. In addition, they are vesicles of endosomal origin [33], formed by a series of processes involving the endosomal sorting complex required for transport (ESCRT) [37] and multivesicular bodies (MVBs) [39]. The first step in the formation of exosomes is inward budding, which creates a membrane-bound internal vacuole. Once that process is completed, the ESCRT facilitates the development of the vacuoles into early endosomes. This is then followed by a second invagination step of vesicles into the endosomes, where they accumulate and mature into MVBs. These MVBs can either be transported to lysosomes destined for degradation or they can fuse with the plasma membrane [40, 41] to be released into the extracellular space, upon which they are referred to as exosomes [21, 38]. Exosomes carry specific protein and RNA cargo, such as heat shock proteins, tetraspanins, and integrins [33, 41, 42]. They are enriched in cholesterol, sphingomyelin, and ceramide, which allow them to be involved in diverse biological and physiological processes, such as coagulation, antigen presentation, and cell signaling and growth [30, 42, 43].

Given the differences between the three main types of secreted vesicles, it is essential that a standard nomenclature and definitions are developed in order to avoid study-to-study variations and possible misinterpretation of data. The International Society of Extracellular Vesicles (http://www.isev.org/) and ExoCarta (http://www.exocarta.org/) have made efforts toward establishing a standardized nomenclature for the different types of vesicles [44].

## 3. Mechanisms Underlying MP Shedding

**Figure 1:**
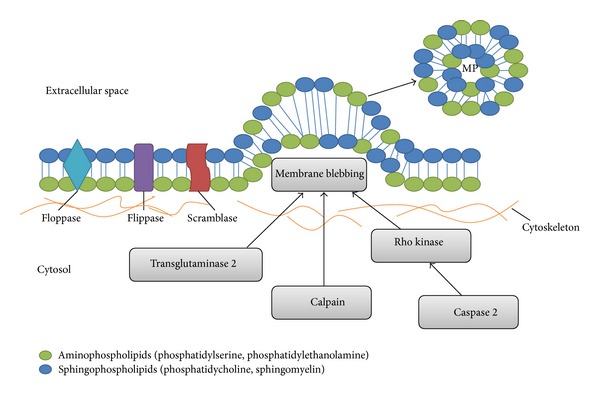
Possible mechanisms responsible for microparticle blebbing and release. Under normal conditions, the plasma membrane is well-structured and characterized by asymmetric lipid distribution. During MP formation, lipid asymmetry is lost, and aminophospholipids are redistributed to the outer leaflet. Cytoskeletal rearrangement induced by caspase 2/Rho kinase, calpain, or transglutaminase 2 results in outward blebbing of the plasma membrane with subsequent MP formation and release. Adapted from Burger et al. [31]; Distler et al. [55].

Throughout their life cycle, cells are continuously subjected to a variety of stimuli that can induce many different signaling cascades and biological responses, including plasma membrane shedding. The shedding process results in the formation of MPs that contain cell membrane constituents and cytoplasmic contents [45]. Thus, MPs can successfully outlive a dying “parent cell”. Although the exact mechanism underlying MP shedding is not yet fully understood, it appears to be a complex process involving cytoskeletal rearrangement and alterations in phospholipid symmetry (Figure 1).

Although resting cells show a constitutive release of MPs [13, 43], activation and apoptosis appear to be the major triggers for the generation of increased quantities of MPs. In addition, certain types of stress such as hypoxia or irradiation, oxidative injury, and shearing stress can increase the number of released MPs [4, 14]. Apart from cell stressors, several groups have also identified specific stimuli that trigger the formation of MPs from different cell types (reviewed by [32]). Platelets, for example, can be induced to shed MPs by exposing them to lipopolysaccharide (LPS), Shiga toxin [46], thrombin [47], collagen [48], interleukin (IL)-6 [49], and erythropoietin [49], just to name a few. Calcium ionophore A23187-induced increase in intracellular calcium [4, 43] triggers MP shedding from platelets, dendritic cells, monocytes, and microglia [50]. In addition to calcium, proinflammatory mediators stimulate monocytes; tumor necrosis factor (TNF)-*α* [51] significantly induces MP formation, as does LPS [52].

Endothelial cells and leukocytes represent a significant source of circulating MPs. Both these cell types respond to TNF-*α* stimulation [53–55], while endothelial cells additionally can be induced to release MPs by IL-1*α* [56], C-reactive protein (CRP) [57, 58], and LPS in the presence of the omega 3 fatty acid docosahexaenoate [59]. Recent in vivo data indicate that retrograde shear stress (“backward” blood flow) can induce endothelial cell-derived MPs (EMPs) in healthy humans [60]. Formation of MPs from other cell populations is less studied, but, as previously mentioned, there is evidence that this process might be universal and that nearly all cell types shed MPs.

The precise molecular mechanisms by which MPs are shed from the plasma membrane remain to be fully elucidated. It is known that cytoskeletal reorganization and plasma remodeling are required for MP formation and shedding. Actin filament dynamics play an important role in the process, as was demonstrated by several studies that used actin polymerization inhibitors cytochalasin D, latrunculin B, and jasplakinolide. Administration of these inhibitors resulted in an increase in MP formation from platelets, megakaryocytes, and T cells [55, 61, 62]. Other studies found that inhibiting calpain, a Ca^2+^-dependent protease, which cleaves the cytoskeletal proteins talin and *α*-actin, accelerates the formation of MPs from platelets [63] and neutrophils [64]. According to Yano et al. [63], calpain exerts its effects in the early stages of MP formation. Other groups have implicated the involvement of the myosin light-chain kinase (MLCK) in the formation of MPs by demonstrating a decrease in MP release when the rat pheochromocytoma PC12 cells were exposed to the MLCK inhibitors KT5926, ML-7, and ML-9 [65]. The same group was one of the first to demonstrate the involvement of Rho signaling in the shedding mechanism.

Since then, other studies have shown that Rho-associated kinase I (ROCK-I), an upstream regulator of MLCK, is involved in cortical myosin-II contraction and the detachment of the plasma membrane from the cytoskeleton, which leads to the release of the MPs [32, 66]. Coleman et al. [66], demonstrated the involvement of ROCK-I by inhibiting its activity with a small molecule inhibitor Y27632. They observed a decrease in myosin light-chain phosphorylation, as well as a decrease in MP formation. The involvement of ROCK-II and caspase 2 in thrombin-induced shedding of EMPs has also been demonstrated [67].

Another enzyme that governs cytoskeletal reorganization is transglutaminase-2. It specifically catalyzes protein cross-linking and has recently been shown to be involved in MP release from smooth muscle cells [68]. Thus, it is apparent that the machinery necessary for MP formation consists of a multitude of factors, which may vary between different cell types.

Externalization of PS is another key aspect of MP formation. The two leaflets of the plasma membrane have distinct compositions. The aminophospholipids, which include PS and phosphatidylethanolamine, are mostly found in the inner leaflet of the cell membrane, whereas phosphatidylcholine and sphingomyelin are found in the external leaflet [45, 69]. The asymmetric distribution of lipids is maintained by three groups of enzymes with very specific roles: flippases, floppases, and scramblases [3, 14, 70].

Most studies report that the surface exposure of PS is an early sign of cell activation or apoptosis, which precedes MP release [3, 29]. Strong support for this derives from studies on individuals with Scott syndrome. They have an impaired ability to externalize PS, which leads to impaired coagulation [71]. In addition, they exhibit reduced MP shedding from platelets [72]. There are, however, other studies that have reported that PS is not externalized in certain MP populations. Based on the absence of annexin-V binding, Horstman et al. [70], observed that only a fraction of the EMPs were in fact PS positive. Annexin-V is a protein that binds to exposed PS thereby allowing its use for MP detection. Interestingly, the MPs from activated endothelial cells are rarely annexin-positive, in contrast to MPs from apoptotic endothelial cells [70]. Another study on monocytic cells concluded that MPs should not be defined based solely on PS expression as this would lead to the exclusion of a large percentage of MPs [73]. At this point, however, the mechanism behind the appearance of PS-negative MPs remains unclear, and biological importance of this phenomenon should be assessed by future studies.

Recently, it has been shown that cell types expressing the purinergic P2X_7_ shed MPs from their surfaces via a specialized mechanism that is dependent on the activation of this receptor by adenosine triphosphate (ATP), leading to the hydrolysis of sphingomyelin to ceramide. Furthermore, this mechanism appears to involve activation of ROCK and p38 mitogen-activated protein kinase (MAPK) [29]. P2X_7_ is an ATP-gated ion channel, highly expressed in immune cells, particularly macrophages, mast cells, and microglia. It can act as a selective ion channel or as a nonselective pore. The latter usually results in apoptosis and cell death. Turola et al. [29] found that MP shedding from these particular cell types is controlled by acid sphingomyelinase (A-SMase), which hydrolyzes sphingomyelin to ceramide. Following P2X_7_ receptor activation, p38 MAPK is phosphorylated; this in turn induces the translocation of A-SMase to the outer leaflet, generating ceramide from sphingomyelin, and thereby inducing the budding of the MPs. The exact mechanism by which ceramide induces budding is still unknown, but it is assumed that it affects membrane fluidity. After being formed, ceramide redistributes within the lipid bilayer, and due to its negative curvature, it causes plasma membrane protrusions. These would in turn contribute to membrane destabilization and facilitate MP shedding [74, 75]. It has become apparent that the formation of MPs is not a uniform process but rather one that involves different mechanisms and is dependent on a variety of factors, including the type of cells and their functional status (activated versus apoptotic).

## 4. Composition of Microparticles

MPs have been identified in human plasma, urine, saliva, and CSF. MPs have been shown to be released by platelets, macrophages, monocytes, B and T cells, neutrophils, erythrocytes, endothelial cells, epithelial cells [61, 76–78], and almost all brain cell types including neural progenitors, neurons, microglia, astrocytes, and oligodendrocytes [10, 19, 74]. As a result, the MPs differ in composition depending on the cell of origin often due to the differences in composition of the acquired membranes. Recent studies have shown, however, that even MPs originating from one single cell type are not always alike [60].

This was first observed in a study on EMPs. Using endothelial cell-specific biomarkers for EMPs, Jimenez et al. [79] demonstrated that the counts of EMPs positive for specific markers varied depending on the stimulus applied. They showed that EMPs released from microvasculature endothelial cells expressed different biomarkers depending on whether the endothelial cells had been undergoing apoptosis or activation induced by TNF-*α*. They concluded that phenotypically distinct populations of MPs were released by the endothelial cells [79].

An additional study conducted by Bernimoulin et al. [73] showed that differential stimulation of human THP-1 monocytic cells resulted in distinct populations of MPs. They stimulated THP-1 cells with LPS or P-selectin and discovered that the resulting MP populations all shared a similar size distribution and a cytoskeletal organization, as well as an antigen expression pattern. Of the 100 proteins that were shown to be common to all MPs, most were cytoskeletal proteins, such as *β*-actin and *α*-actinin 4. The proteins CD18, CD81, and CD45 were also common to all populations. The differences between the populations were mainly in the degree of PS expression. P-selectin-induced MPs had fewer PS-positive MPs compared to the LPS-induced MPs. Additionally, leukocyte-associated immunoglobulin-like-receptor-1 (LAIR-1), a cell surface protein, was only found in MPs derived from the P-selectin-stimulated cells. This led Bernimoulin et al. [73] to hypothesize that the translocation of PS from the inner to the outer membrane is regulated differently depending on the cell stimulus. The resulting differences in MP composition may affect the biological roles they play in homeostasis, inflammation, and immune regulation.

**Figure 2:**
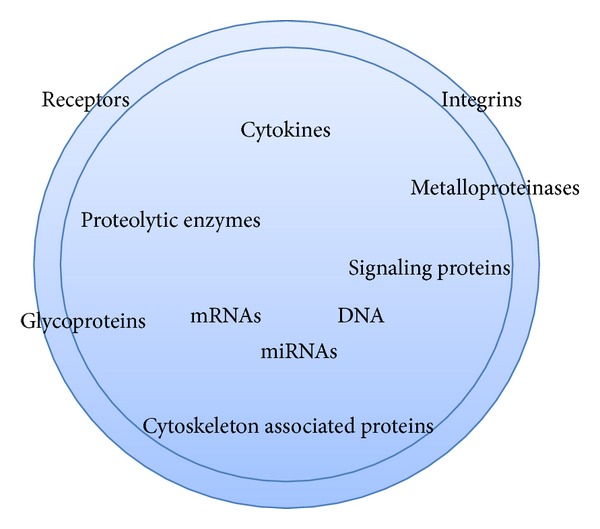
Microparticles as a storage pool for a variety of bioactive molecules. Their content varies depending on the cell of origin and the inducing stimulus.

Apart from having different surface markers, MPs are also considered to be storage pools of diverse bioactive molecules [45, 80] (Figure 2). Their content may include proteins (e.g., signaling molecules, receptors, integrins, and cytokines), bioactive lipids, nucleic acids (e.g., miRNA, mRNA, DNA), and organelles [81–83]. MPs from tumor cells, neutrophils, and astrocytes are enriched with metalloproteinases and other proteolytic enzymes; such MPs aid in the digestion of the extracellular matrix, which accompanies inflammation and tumor invasion [43, 84]. MPs from microvascular endothelial cells also contain matrix metalloproteinase (MMP) 1, MMP2, MMP7, and MMP13, which bind and degrade fibronectin [85]. MPs from human atherosclerotic plaques contain an active form of a human disintegrin and metalloproteinase 17 (ADAM17), which can induce the release of the proinflammatory cytokine TNF-*α* [86]. Platelet MPs carry integrins, such as the plasma membrane glycoproteins GPIb, GPIIb-IIIa, and P selectin [45], which are important for coagulation. It is apparent that the MP content varies according to the cell type MPs are derived from and the expected biological function of the individual MP. Recent studies have also identified miRNA in MPs [32, 82]. The MP membrane protects miRNAs from degradation by RNases and allows for their effects to be exerted on target cells at much greater distances.

## 5. Methods Used for Isolating and Detecting Microparticles

Increasing evidence supporting the involvement of MPs in the pathogenesis of a variety of diseases [8, 16] has led to recent expansion of MP research. There is particular interest in using circulating MPs, found in the blood and other body fluids, as predictive and diagnostic biomarkers. Therefore, it is important to refine and standardize the methods used to isolate MPs in the research and clinical laboratory setting. There are still differences between isolation protocols used by individual laboratories; however, a standard method of isolation is being developed. Müller [87] has summarized goals for achieving good quality MP samples that could be used for research and analysis. They include the following. (i) Validation of high-throughput analytical methods that allow for the discrimination between different types of secreted vesicles on the basis of their physical and chemical properties (e.g., size, density, surface receptors, and protein content). Such methods may include mass spectrometry, enzyme-linked immunosorbent assays (ELISA), and reverse transcription-polymerase chain reaction (RT-PCR). (ii) Improvement and simplification of the standardization and calibration procedures for flow cytometry. (iii) Validation of novel membrane-permeable dyes that could be used for fluorescent staining of nucleic acids (DNA, mRNA, and miRNA) to increase the specificity and sensitivity of the staining procedures. The common theme between all the criteria is the development of standard operating procedures to ensure that results obtained in different laboratories are comparable. Established tools being used by most laboratories include flow cytometry and fluorescence microscopy; however, some research groups have begun to develop more precise and sensitive methods for the isolation and analysis of MPs such as nanoparticle tracking analysis [88–90].

### 5.1. Conventional Methods

The essential steps in MP analysis include isolation, detection, differentiation, and quantification. A variety of methods, which all have their own advantages and disadvantages, can be used to accomplish these steps [70, 87, 91].

#### 5.1.1. Filtration

This method represents one of the least expensive, more convenient, and less labor-intensive procedures of MP isolation. It comes with the added benefit of being able to analyze large volumes of samples relatively quickly. The biological sample, such as plasma or cell culture medium, is passed through filters with an appropriate pore size (0.1–1 *μ*m) that are made of materials that do not bind particles nonspecifically [92–94]. It is possible to increase the selectivity of this technique by adding a label specific to the MP of interest. Thus, Grant et al. [92] isolated PS-positive plasma MPs using annexin-V, while Bianco et al. [50] used this method to detect and isolate MPs shed from microglia.

#### 5.1.2. Centrifugation

MPs from biological fluids or cell cultures are more commonly isolated by differential centrifugation. The process is usually comprised of two steps: (i) the cleaning step and (ii) the collection step. The cleaning step involves spinning the samples at low speeds for a short period of time with the purpose of removing intact and broken cells, cell debris, and large cellular organelles. It is important to keep in mind, however, that smaller fragments and debris might still be present along with the isolated MPs, which is why further methods such as immunostaining need to be applied in order to confirm the presence of MPs. As the centrifugation process has not been standardized as of yet, the centrifugation speeds and times used vary between different laboratories. In some cases samples are initially centrifuged at 1,500 ×g for 15 min [91], while others recommend centrifugal forces between 200 and 300 ×g for 5 min [78, 87]. The collection step is usually completed at medium centrifugal forces for an intermediate length of time (e.g., 10,000–16,000 ×g for 10–20 min) [78, 91]. If ultracentrifugation force is reached (e.g., 100,000–150,000 ×g), it becomes likely that the vesicles isolated would also include exosomes, as they are smaller than MPs and sediment at such higher centrifugation force [28, 87, 95]. Therefore, it is important to use appropriate centrifugal forces in order to avoid mixed populations of membrane vesicles, as this may alter the results obtained. This point could be illustrated by the conflicting results obtained by two different studies that investigated the procoagulant activities of MPs from sickle cell disease patients [96, 97]. These studies used significantly different centrifugation conditions to prepare their samples: 18,890 ×g for 30 min [96] versus 100,000 ×g for 60 min [97], yet both called the isolated particles MPs.

#### 5.1.3. Electron Microscopy

This method has been used in numerous studies to classify MPs [70, 98, 99]. The high resolution of electron microscopy allows for the determination of the size and morphology of MPs in great detail [100]. However, it does not permit quantification of MP samples. Furthermore, when comparing images obtained by different research groups, it becomes apparent that a high degree of MP heterogeneity exists [101, 102]. Another technique that provides good quality data on size measurements and morphology is atomic force microscopy. It characterizes nanoscale objects with very high resolution, thus providing structural details that were previously unknown [103].

#### 5.1.4. Fluorescence Microscopy

Most cells and MPs do not exhibit an intrinsic fluorescence, which is why they are first treated with a fluorescently labeled antibody or proteins bound to fluorophores [40]. The most commonly used fluorophores are organic dye molecules and quantum dots. One study used quantum dots and fluorescence microscopy to monitor membrane fusion and retrieval [104]. The intensity of the fluorescent signal detected does not necessarily correlate with the volume of the MPs from which the signal is being emitted. Therefore, the size of individual MPs cannot be determined, but it is possible to gain information on the concentration of MPs present [40].

#### 5.1.5. Flow Cytometry

Flow cytometry is one of the most common methods used for identification and quantification of MPs. Most modern flow cytometers can count, separate, and isolate particles at a rate of thousands per second based on specific properties (e.g., size) or biomarkers present on the particle surface. Light scattering is commonly used to determine sizes of larger cell types, but it is less suited for determining the size of particles that are smaller than 300–500 nm [105]. The use of fluorescent signals instead of visible light may provide a solution to this problem. Fluorescence intensity is higher than light-scattering intensity for nanometer-sized particles [40], thus increasing the counting efficiency for MPs of these particular sizes.

**Figure 3:**
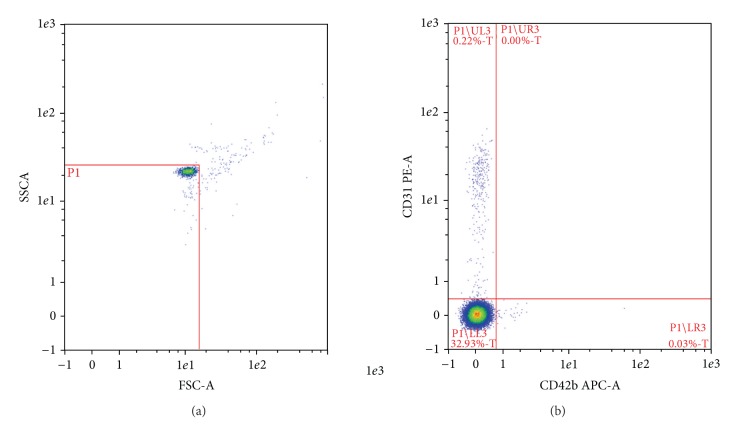
Identification of microparticles by flow cytometry based on particle size and surface protein expression. (a) Traceable beads of a defined size, in this case 900 nm (Nanobead NIST Traceable Particle Size Standard, Polysciences Inc., Warrington, PA, USA), were used to define microparticles in the P1 gate. (b) Endothelial microparticles are identified in platelet poor plasma as CD31+/CD42b− events in the upper left quadrant within this P1 gate. Methods are based on those described by Jenkins et al. [60].

Molecular stains that have been used in conjunction with flow cytometry are annexin-V and more recently biomaleimide. Enjeti et al. [91] introduced the use of biomaleimide due to its ability to bind biological membranes via cysteine residues and thiol groups in proteins, as well as its fluorescent nature. They used it to measure total circulating MP content in human plasma and found that it is a more cost efficient technique yielding results comparable to those obtained with annexin-V [91]. They concluded that biomaleimide could provide a good alternative screening technique for the detection of MPs. Other studies have been using lipid markers such as calcein AM or PKH67 in conjunction with annexin-V to ensure that a significant percentage of MPs does not remain unstained, which would underestimate the MP concentrations present [14, 73, 76]. Like all the other methods mentioned, flow cytometry procedures need to be standardized to minimize study-to-study variability [106]. One approach that can be used is to calibrate using beads of a predetermined size (500–1,000 *μ*m) and to use fluorescent probes that bind to specific cell surface proteins indicative of activation or apoptosis to define distinct MP populations. This technique has been successfully applied in our laboratory with MPs isolated from THP-1 monocytic cells, platelets, and endothelial cells. The cell surface markers CD31 and CD42b, which are specific for platelets and endothelial cells, were used in combination with the calibration beads to identify and determine particles <900 *μ*m in size (Figure 3).

### 5.2. Alternative Methods

A variety of new methods have been developed recently for the detection and isolation of MPs based on the newly acquired information on the diversity of nucleic acids associated with MPs. MPs have been shown to carry mRNA, miRNA, and non-coding RNA (ncRNA), as well as DNA [6, 44, 107]. As a result, fluorescent probes and chromophores directed against the MP-associated nucleic acids have been employed to detect MPs.

Nanoparticle tracking analysis (NTA) is another technique, which measures the absolute size distribution of MPs between 50 and 1,000 nm in addition to quantifying the MPs in the sample [108]. This method relies on the correlation between the Brownian motion of particles in fluid and the light scattering properties of a laser beam [87, 90]. This method is less labor-intensive, which allows for higher throughput analysis and, more importantly, it has a lower size detection limit than flow cytometry (~50 versus ~300 nm). Several studies have successfully applied this technique for the detection of MPs in plasma and in the supernatant of cultured cells [88, 89].

## 6. Microparticles in the CNS

MPs can contribute to intercellular communication without direct cell-to-cell contact. A number of studies have demonstrated the involvement of MPs in neuronal development, synaptic activity, and nerve regeneration [21]. Most cell types of the CNS, including neurons [109], astrocytes [95], and microglia [50], have been shown to release membrane vesicles. Microglial cells, in particular, are of interest, as they are the resident macrophages of the CNS and are recognized as the essential components of the intrinsic brain immune response [1]. Microglia therefore may act as a source of MPs in the CNS, which may have implications for certain CNS pathologies.

### 6.1. Development

**Figure 4:**
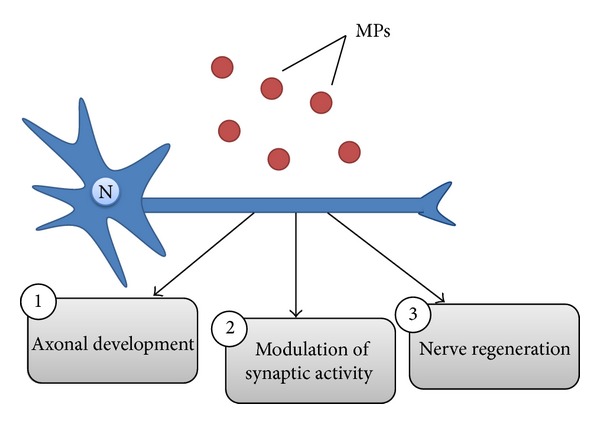
Effects of microparticles on neuronal cells. (1) Spatial and temporal gradients of MPs can contribute to axonal growth. (2) Specific MP proteins released within the synapse can affect synaptic function. (3) The transfer of MPs containing ribosomes and mRNA from Schwann cells to the injured nerves can promote protein synthesis and regeneration.

Increased levels of neural stem cell-derived MPs that contain the stem cell marker prominin-1 (CD133) can be measured during neurogenesis in developing mouse brains [19, 83]. Although the exact relationship between the released MPs and neural differentiation is still unclear, two hypotheses exist that attempt to define the physiological roles of these MPs. First, prominin-1 is known to interact with membrane cholesterol and lipid rafts [83]. As lipid rafts are actively involved in signal transduction, MPs that originate from stem cells may carry the determinants necessary for cell differentiation. Secondly, the prominin-1-positive MPs may participate in intercellular communication [83]. Other studies suggest that these MPs transfer mRNAs encoding pluripotent transcription factors, which can affect the phenotypes of other cells [4]. In addition, MPs are essential for establishing the spatial and temporal gradients critical in development. In support of this, MPs were shown to be involved in the transfer of *β*-galactosidase from neuronal floor plate cells to neighboring axons, thereby contributing to axonal path finding [110] (Figure 4). In terms of temporal patterning, oligodendrocytes release MPs to suppress myelination until they receive the appropriate signals from neurons indicating that maturation is complete [111].

### 6.2. Synaptic Activity

MPs have also been shown to participate in synaptic activity. The first studies were conducted on exosomes, which showed that upon depolarization, undifferentiated cortical neurons released exosomes containing L1, a neuronal cell adhesion protein, and the GLUR2/3 subunit of *α*-amino-3-hydroxy-5-methyl-4-isoxazolepropionic acid (AMPA) receptors [112]. A recent study by Antonucci et al. [75] confirmed that MPs can modulate synaptic activity (Figure 4). MPs released from microglia may act on the presynaptic site of the excitatory synapse, increasing the neurotransmitter release probability, and consequently increasing synaptic activity and excitatory transmission in neurons [75]. The researchers found a concentration-dependent increase in the release of glutamate from neurons in conjunction with increases in the miniature excitatory postsynaptic current (mEPSC) frequency. In addition, they demonstrated that MPs induced sphingolipid metabolism in neurons; sphingosine and its metabolite sphingosine-1P facilitate transmitter release from synaptic terminals [75].

### 6.3. Nerve Injury and Regeneration

MPs may serve a protective role in the CNS and are involved in mechanisms that are activated after nerve damage [113]. Schwann cells that surround a damaged nerve release MPs containing ribosomes that transfer their content to the damaged axon [21, 114]. In addition, the MPs can deliver mRNAs to the injured neurons in order to stimulate proliferation and protein synthesis needed for regeneration (Figure 4). Frühbeis et al. [115] demonstrated that exosomes may represent a novel mode of glia-neuron communication contributing to maintenance of neuronal integrity. They showed that glutamate triggers release of exosomes by oligodendrocytes. These exosomes, along with their protein and RNA cargo, are then internalized by neurons. Under conditions of cell stress, the cargo was shown to convey protection and improve neural viability [115]. During brain injury, however, MPs can contribute to the exacerbation of the injury. The resulting increases in extracellular ATP may lead to the release of microglial MPs containing the proinflammatory cytokine IL-1*β*, which is a key regulator of neuroimmune responses [29, 81]. Therefore, upon stimulation, microglia release MPs, which act as the amplifying agents of inflammation.

## 7. Microparticles and Disease

Low levels of MPs can be detected in the blood and body fluids of healthy individuals [156, 157]. The levels of different types of MPs are primarily determined by the rate of formation versus the rate of their clearance. Clearance is mainly achieved by the action of proteases and phospholipases, which directly degrade the MPs. Other clearance mechanisms involve the action of different resident macrophages, such as the liver Kupffer cells [158] and the lung macrophages [159], which take up the circulating MPs in a PS-dependent manner. Splenocytes can also phagocytose MPs in order to clear them [160]. Researchers have found that the size of a MP affects its clearance. Litvack et al. [161] found an inverse correlation between particle size and IgM-mediated clearance by macrophages, showing that IgM promotes the clearance of smaller sized particles, including MPs (<1 *μ*m) compared to particles over 1 *μ*m in diameter.

In individuals with certain pathological states, the MP levels differ from the baseline concentrations found in their healthy counterparts; the concentration of MPs could be either elevated or decreased (see Table 2). Therefore, MPs may play an important role in the development, progression, or resolution of a wide range of diseases including different cancers, infectious diseases, autoimmune diseases, cardiovascular diseases, and inflammatory diseases [20, 117, 162, 163].

Factors that regulate MP release or their clearance during disease progression are complex and remain to be elucidated. In pathological states such as pulmonary hypertension, intracerebral hemorrhage, endotoxemia, and hepatitis C, increased levels of MPs are usually correlated with a more severe disease progression and adverse outcomes [136, 144, 147, 164]. Although most disorders are characterized by higher counts of MPs, there are diseases that exhibit decreased or unchanged MP levels regardless of the severity of the disease. Steppich et al. [141] found that the levels of MPs in patients suffering from deep vein thrombosis were not increased compared to the control group. Other diseases that exhibit a similar phenomenon include certain tumors, such as gynecological, gastric, colorectal, and brain cancers [118, 165], as well as some nephropathies, including nephrosclerosis [77]. The observed discrepancies between the different pathologies might be a result of the diverse types of MPs that are released and differences in MP isolation techniques used. It is possible that only certain subtypes of MPs (endothelial, platelet, and neuronal) are significantly affected during the progression and resolution of a particular disease. So far very few studies have attempted to evaluate this possibility; nevertheless it is feasible that analysis of MP concentration and composition of MP population could be used to improve the detection of different pathologies. This is especially important in neurodegenerative diseases, as it is often particularly difficult to accurately establish the early stages of these disorders [10].

### 7.1. The Role of Microparticles in CNS Pathologies

The CNS cells (neurons, astrocytes, oligodendrocytes, and microglia) are subjected to different types of stress, which can lead to MP shedding. It is becoming increasingly evident that MPs can contribute to the onset and progression of some neurodegenerative and neuroinflammatory diseases [10, 50, 98]. This assumption is mainly based on two observations. First, MP numbers are increased in some CNS diseases, and secondly, MPs derived from patients affected by CNS disorders often carry inflammatory mediators and other bioactive molecules on their surface [98]. The MPs can be isolated from the plasma or the CSF of patients suffering from several different CNS diseases, which gives hope that they can be used as biomarkers for these diseases allowing earlier detection and monitoring of the progression of diseases. In addition, some researchers have even suggested using MPs themselves as therapeutic agents due to the specific cargo that they might carry. A recent study showed that platelet-derived MPs carry a variety of growth factors. Furthermore, when applied to neural stem cells after brain injury, such MPs have the ability to promote neurogenesis by stimulating neural stem cell proliferation, migration, and differentiation [166].

#### 7.1.1. Multiple Sclerosis (MS)

Traditionally, MS has been thought of as an autoimmune disease, in which the body's T cells recognize a component of myelin as foreign and initiate an auto-destructive process within the CNS. In addition to demyelination, MS is characterized by the presence of inflammatory white and gray matter lesions in the brain and spinal cord [167]. More recent evidence suggests that MPs may also contribute to the pathogenesis of MS. This is in part due to the presence of MPs in the CSF of MS patients. Initial evidence provided by Scolding et al. [24] demonstrated the presence of MPs bearing both the membrane attack complexes and galactocerebroside, which had important implications for the nature of MS. More recently, Verderio et al. [20] confirmed the presence of increased levels of myeloid-derived MPs in the CSF of relapsing-remitting MS patients. By using experimental autoimmune encephalomyelitis (EAE), the animal model of MS, the authors investigated the efficacy of the clinically available oral MS drug FTY720. They found that FTY720 treatment significantly decreased the levels of MPs in the CSF of EAE mice [20].

Studies by Minagar et al. [23] and Fauré et al. [112] also showed that levels of EMPs correlated closely with the disease progression in MS. Both groups confirmed that endothelial cell dysfunction contributed to MS and that MPs could be used as evidence for this dysfunction, as they expressed specific markers for blood-brain barrier (BBB) damage in MS. These markers included CD51 and platelet-endothelial cell adhesion molecule-1 (PECAM-1 or CD31). CD51-containing MPs were chronically elevated in MS patients regardless of disease exacerbation or remission, while CD31-containing MPs were increased during exacerbation but decreased during remission [23, 112]. The authors further suggested that CD31-positive MPs indicate acute injury to the endothelium (i.e., exacerbation), while CD51-positive MPs indicate chronic injury to the endothelium. Therefore, analysis of MP composition may help with decisions on the MS treatment options after diagnosis. The levels of CD31-positive MPs were also used to determine the effectiveness of interferon (IFN)-1*β* 1a treatment in relapsing-remitting MS [168]. Lowery-Nordberg et al. [169] found that plasma levels of CD31-positive and CD54-positive MPs may serve as effective biomarkers allowing for the assessment of the effectiveness of IFN-1*β* 1a treatment. Levels of both types of MPs significantly decreased with the treatment. Furthermore, lower MP levels were associated with a decrease in the number and volume of MS lesions present [169].

Other studies have looked into the role that EMPs play in MS progression, focusing on specific disease mechanism [152, 170]. Both these studies showed that the EMPs form complexes with monocytes, which facilitate the transendothelial migration of these cells through the BBB. One of the studies, in particular, found that the monocyte migration could be inhibited by IFN-1*β* 1b [111], which may represent a molecular target for future treatment options.

#### 7.1.2. Cerebral Malaria

Cerebral malaria occurs in 1 to 8% of* Plasmodium falciparum* infections and is often fatal. EMPs have been shown to be increased in patients with severe cerebral malaria complicated with coma compared to uncomplicated malaria or healthy controls [171]. The parasite-derived products activate platelets and induce TNF-*α* production by monocytes, which in turn promotes EMP shedding from endothelial cells. EMPs have both proinflammatory and prothrombotic properties [3]. Jimenez et al. [79] further elucidated the mechanism of action of MPs in cerebral malaria. In a mouse model, they demonstrated that ABCA1, a membrane transporter regulating the transbilayer distribution of PS at the outer leaflet of the plasma membrane, contributes to the pathogenesis of cerebral malaria by affecting MP shedding. ABCA1 knockout mice showed decreased PS externalization, as well as low circulating MP levels, which led to their complete resistance to cerebral malaria [83]. These results directly implicate MPs in the pathogenesis of cerebral malaria and indicate that ABCA1 could be used as a target for therapeutic interventions.

#### 7.1.3. Alzheimer's Disease (AD)

Alzheimer's disease is the most common progressive form of fatal dementia in humans [172]. The two major hallmarks of AD are senile plaques and neurofibrillary tangles in the brain, which were first described over 100 years ago [173]. The plaques are caused by the abnormal accumulation of amyloid-*β* peptide (A*β*), which is released from an amyloid precursor protein (APP) upon limited proteolysis [174]. The neurofibrillary tangles are mainly composed of a cytoskeletal microtubule-associated protein, called tau, which becomes hyperphosphorylated, dissociates from the microtubules, and consequently self-aggregates in the cytosol [46]. There is increasing evidence that MPs may be involved in the transfer of A*β* between cells [98, 167], as well as in the active secretion of tau protein in the brain [17, 175].

MPs have been shown to actively bind and transport APP [176] and soluble A*β* [167]. Soluble A*β* is released by activated platelets and carried within MPs in healthy subjects [167]. The platelet A*β* is necessary for normal platelet function and coagulation. Studies have confirmed that A*β* peptides are actively released from platelets and that the released A*β* further activates more platelets, which may initiate a vicious cycle of increased platelet activation and A*β* release leading to the development of cerebral amyloid angiopathy [46]. Platelets play a key role not only in hemostasis but also in inflammatory processes, as they secrete a wide variety of potent inflammatory mediators including chemokines, cytokines, and prostaglandins [177]. Therefore, the uncontrolled activation of platelets in AD patients can lead to a chronic state of inflammation causing endothelial stress, and MP shedding along with A*β* release. Increased transport of MP-associated A*β* around the body and to the brain can possibly contribute to increased amyloid deposition [168].

The M1C neuroblastoma tauopathy model and immunoblotting analysis were used to confirm the presence of tau within MP fraction. It was also possible to detect increased levels of MP-associated phosphorylated tau protein in the CSF of patients suffering from mild cognitive impairment or early AD compared to healthy control subjects [48, 178]. This is a very promising finding and may provide a method for early diagnosis of AD by measuring MP content in CSF.

In light of the evidence showing MP involvement in the pathogenesis of AD, there has been increasing debate about possible prion-like activity of MPs in AD. The possibility that A*β* and tau aggregates may be transmissible, similar to prions, is becoming increasingly popular among research groups [168, 179, 180]. This hypothesis stemmed from experiments conducted in transgenic mice expressing human A*β* [181]. They demonstrated that intracerebral injections of brain extracts from amyloid plaque-containing brain tissue from AD patients into the transgenic mice resulted in increased amyloid plaque formation. This indicated that the A*β* aggregates could be capable of self-replicating in susceptible hosts, which is similar to the characteristics of a prion. These observations have since been reproduced in other murine models of AD [182, 183]. Consequently, MPs might be involved not only in the active release of the pathogenic factors but also in the spread of the neurodegenerative disease process. Joshi et al. [178] demonstrated that MPs derived from AD patients were directly toxic to cultured neurons. They found that this neurotoxicity was due to their ability to promote solubilization of A*β* fibrils to neurotoxic soluble species. This observation indicates that MPs may not only act as transporters of neurotoxic factors but could also actively contribute to the progression of AD. Further supporting evidence is required, however, to determine whether MPs act mainly as a transport mechanism or whether their structural and molecular make up contributes significantly to the disease pathology.

## 8. Conclusion

Originally believed to be inert by-products of platelet activation, MPs have emerged as key mediators of intercellular communication and protective mechanisms in the CNS, as well as biomarkers of disease. The latter aspect concerning MPs is of greatest interest, as they may be used for the development of new diagnostic assays directed at identifying the early stages of certain diseases and response to therapy. This will be particularly valuable for the CNS diseases, which typically cannot be diagnosed early. In healthy individuals, MPs are involved in axonal development, modulation of synaptic activity, and nerve regeneration, but certain MPs, particularly EMPs, have also been shown to be associated with the onset and progression of a variety of diseases, yet the mechanism underlying this change in the roles that MPs play remains unclear.

The challenge facing future research will be the optimization and standardization of the preanalytical handling of samples. As the methods for isolating and characterizing MPs improve and advance, however, it will allow for a better understanding of the mechanisms underlying formation and composition of different types of MPs. Flow cytometry, as well as some of the newer alternative methods, will be critical for detailed characterization of MPs. This will provide essential information on the biological effects of MPs and expand the current knowledge on the physiological and pathophysiological roles that they play. And finally, MPs may also have a potential as a novel class of therapeutics due to their ability to transport bioactive molecules (reviewed by [184]).

## Figures and Tables

**Table 1:** Physical properties of membrane-derived vesicles.

	Exosomes	Microparticles	Apoptotic bodies
Size	40–100 nm	100–1000 nm	>1000 nm
Appearance	Homogeneous	Heterogeneous	Heterogeneous
Sedimentation	100,000 g	10,000 g	1,200 g
Site of origin	Multivesicular bodies (MVBs)	Plasma membrane	Cells undergoing apoptosis
Lipid composition	Cholesterol, ceramide	Phosphatidylserine, cholesterol	Phosphatidylserine
Main protein markers	Tetraspanins, GPI-proteins	Integrins, selectins, CD40 ligand	Histones

Adapted from Burger et al. [31]; Cocucci et al. [43]; Théry et al. [28].

**Table 2:** Changes in MP levels associated with a variety of disorders.

	MP levels	References
Cancer		
Acute promyelocytic leukemia	↑promyelocytic-derived MPs	[116]
Brain cancer	↑tumor-derived MPs	[117–119]
Breast cancer	↑endothelial-derived MPs ↑leukocyte-derived MPs ↑platelet-derived MPs	[120]
Colorectal cancer	↑platelet-derived MPs	[118, 121]
Gastric cancer	↑platelet-derived MPs	[122]
Lung cancer	↑monocyte-derived MPs	[123]
Prostate cancer	↑platelet-derived MPs	[124, 125]

Autoimmune disorders		
Crohn's disease	↑endothelial-derived MPs ↑platelet-derived MPs	[126, 127]
Diabetes mellitus (type 2)	↑monocyte-derived MPs ↑endothelial-derived MPs ↑platelet-derived MPs	[128–132]
Rheumatoid arthritis	↑granulocyte-derived MPs ↑monocyte-derived MPs ↑platelet-derived MPs	[18, 133, 134]
Neuropsychiatric systemic lupus erythematosus (NSLE)	↓monocyte-derived MPs in active NSLE	[135]

Infectious disease		
Hepatitis C	↑T lymphocyte-derived MPs	[136]

Cardiovascular diseases		
Coronary syndromes	↑endothelial-derived MPs	[137–139]
Hypertension	↑endothelial-derived MPs ↑monocyte-derived MPs ↑platelet-derived MPs	[129, 140]
Thrombotic disorders	MP levels unchanged	[141]
Myocardial infarction	↑endothelial-derived MPs ↑platelet-derived MPs	[142]
Preeclampsia	↑endothelial-derived MPs	[143]
Pulmonary hypertension	↑endothelial-derived MPs ↑leukocyte MPs-derived	[144]

Inflammatory diseases		
Vasculitis	↑endothelial-derived MPs ↑platelet-derived MPs	[77, 145]

CNS disorders		
Alzheimer's disease	↑endothelial-derived MPs	[146]
Basal ganglia hemorrhage	↑platelet-derived MPs	[147]
Cerebral malaria	↑endothelial-derived MPs	[25, 148]
Ischemic stroke	↑endothelial-derived MPs ↑platelet-derived MPs MP levels unchanged	[149–151]
Multiple sclerosis	↑endothelial-derived MPs	[23, 152, 153]
Traumatic brain injury	↑endothelial-derived MPs ↑platelet-derived MPs	[154, 155]
